# Molecular biology for green recovery—A call for action

**DOI:** 10.1371/journal.pbio.3001623

**Published:** 2022-04-22

**Authors:** Marta Rodríguez-Martínez, Jens Nielsen, Sam Dupont, Jessica Vamathevan, Beverley J. Glover, Lindsey C. Crosswell, Brendan Rouse, Ben F. Luisi, Chris Bowler, Susan M. Gasser, Detlev Arendt, Tobias J. Erb, Victor de Lorenzo, Edith Heard, Kiran Raosaheb Patil

**Affiliations:** 1 European Molecular Biology Laboratory, Heidelberg, Germany; 2 BioInnovation Institute, Copenhagen, Denmark; 3 Department of Biological and Environmental Sciences, University of Gothenburg, The Sven Lovén Centre for Marine Infrastructure, Kristineberg, Sweden; 4 International Atomic Energy Agency, Principality of Monaco, Monaco; 5 Department of Plant Sciences, University of Cambridge, Cambridge, United Kingdom; 6 European Bioinformatics Institute (EMBL-EBI), European Molecular Biology Laboratory, Wellcome Genome Campus, Hinxton, United Kingdom; 7 Department of Biochemistry, University of Cambridge, Cambridge, United Kingdom; 8 Institut de Biologie de l’École Normale Supérieure (IBENS), Département de Biologie, École Normale Supérieure, CNRS, INSERM, Université de Recherche Paris Sciences et Lettres (Université PSL), Paris, France; 9 ISREC Foundation Agora Cancer Research Center, Lausanne, Switzerland; 10 Max Planck Institute for Terrestrial Microbiology, Marburg, Germany; 11 Systems and Synthetic Biology Department, Centro Nacional de Biotecnología (CNB-CSIC), Madrid, Spain; 12 MRC Toxicology Unit, University of Cambridge, Cambridge, United Kingdom

## Abstract

Molecular biology has huge potential to help tackle climate change and biodiversity loss but is largely absent from current strategies. Community-wide action is needed to bring molecular biology to the forefront of climate change solutions.

## Teaser

Molecular biology holds a vast potential for tackling climate change and biodiversity loss. Yet, it is largely absent from the current strategies. We call for a community-wide action to bring molecular biology to the forefront of climate change solutions.

Climate change is one of the greatest challenges faced by humankind. Among its many effects, temperature rise and other anthropogenic impacts are causing biodiversity loss at an alarming rate, signalling the onset of a sixth mass extinction. The goal of limiting the global temperature rise to 1.5°C above the pre-industrial level requires an immediate and drastic change of societal and economic organization. While it was reassuring to see that physical and earth sciences were at the core of the COP26 meeting held in Glasgow in October and November 2021, life sciences were underrepresented, and molecular life science was alarmingly absent. In this Perspective, we call for a central role for molecular biology in providing solutions to climate and environmental challenges.

The current focus for climate change mitigation and adaptation is largely on non-biological technologies, such as chemical CO_2_ capture and photovoltaic energy. While nature-based measures, such as those around conservation and agriculture, are receiving increasing attention, the molecular life sciences remain on the fringe, despite their vast untapped potential. Molecular biology, which enables understanding and engineering of life’s fundamental processes from the cellular to the planetary scale, can contribute not only to mitigating and adapting, but also to reverting the current worrying trends of temperature rise and biodiversity loss.

That biological processes can profoundly impact planetary climate is well established. We owe the rich biodiversity on this planet to the massive oxygenation started by microbial photosynthesis circa 2 billion years ago. And, paradoxically, the fossil fuels, the use of which caused the current crisis, are themselves the product of photosynthesis. Although molecular biology has provided great insights into these and other essential processes of life, it is missing from the frontline strategies for climate change solutions. This is concerning, as molecular biology is essential to monitoring ecosystem health, development of optimal intervention strategies, and the invention of tools to implement them.

**Fig 1:**
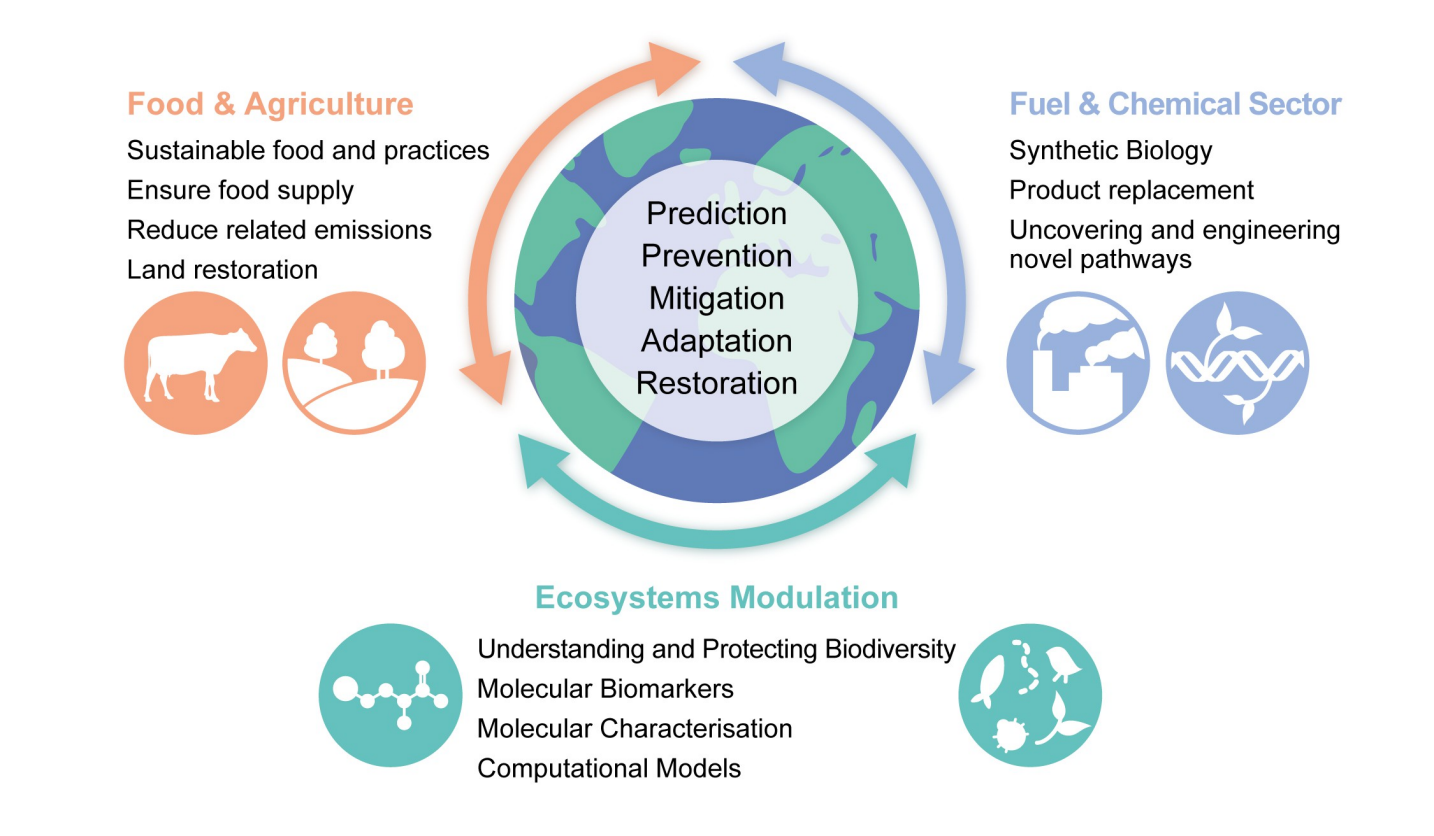
Overview of the main areas wherein molecular biology can make an impact in tackling the current environmental crisis. Molecular biology solutions offer a great potential to tackle the environmental crisis, including that for prediction, prevention, mitigation, adaptation, and restoration. Potential solutions discussed in this Perspective fall under three main categories: Food and agriculture, fuel and chemical sector, and ecosystems modulation. Arrows indicate interconnectivity between the solutions under.

To understand the key areas in which molecular life sciences can make an impact, the European Molecular Biology Laboratory recently hosted a scientific workshop under the All4Climate Italy 2021 programme. Existing and potential solutions were identified (Fig 1), which together span challenges to four planetary boundaries [1,2]: global warming, loss of biodiversity, biogeochemical flows, and manufactured pollutants [3]. Below we outline some of the strategies discussed in the meeting and potential pathways to their realisation.

One of the key areas of potential impact is changes to current food and agricultural systems that can considerably mitigate greenhouse gas emissions [4]. Molecular biology could help through developing novel foodstuffs that provide balanced nutrition while maintaining sensory appeal. Engineering and breeding of crops for, e.g., enhanced nutrition and drought- or salt-tolerance, will be essential to maintain food security. In agricultural practice, modulation of soil, gut and rumen microbiota could help reduce methane emissions from cattle and nitrous oxide emissions from intensified land usage. The balance in global N_2_ flows could be restored by replacing chemical fertilizers with microbial solutions to boost direct N_2_ fixation. In polluted ecosystems, some of which are already pushed beyond their tipping point, engineered, or evolved microbial communities could be deployed to clear the pollutants.

In the fuel and chemical sectors, the vast biochemical diversity of organisms [5, 6] should be tapped for uncovering and engineering novel enzymes and carbon converting pathways. Using these, synthetic biology could help replacing fossil fuels and petrochemical-based materials with renewable resources based on photosynthesis and through valorising waste streams [7, 8].

Another opportunity for impact is a targeted ecosystem modulation. Molecular characterization of inter-species interactions and computational models built on these could help us predict complex ecosystem dynamics and guide interventions to counteract the negative effects of anthropogenic emissions. Plants and environmental microbiomes could be modulated to enhance their capacity to capture CO_2_ and methane. This way, the gap between the carbon sequestering capacities of natural and planted forests could be reduced through model-guided promotion of biodiversity. Similar approaches could halt biodiversity loss in the ocean and promote CO_2_ fixation by phytoplankton and algae, with further capacity boosting through rational genetic engineering [9, 10]. Genomic and metabolomic markers could be developed as biomarkers for monitoring ecosystem health and provide early warnings of tipping points [11]. Integrating these modulation approaches with land usage, marine resources and economic policies would enable better adaptation of local communities to climate change and the implementation of countermeasures for restoring ecological balance.

How can we realize molecular biology solutions? First and foremost, we should aim, by working collaboratively across the globe, to put molecular life science firmly onto the agenda of the United Nations Framework Convention on Climate Change (UNFCCC). The potential of the solutions should be communicated in ways that are accessible to policymakers and the general public. Biologists should be introduced to opportunities in climate science, and climate scientists to the opportunities offered by molecular life science. This interdisciplinary research could be facilitated through creating dedicated programmes and funding schemes. A greater connection between research and industry is also needed, where industrial players are involved early on to help identify the most promising solutions, and to realize their potential by scaling up and integrating them into economic chains. For this to happen, the molecular life sciences need greater focus from governments and industry alike to develop the needed infrastructures and ambitious projects that link molecular and cellular researchers with those investigating planetary health.

Many more and better ideas than discussed here will no doubt emerge as more scientists get involved in this mission. The immediate task is to call for a community-wide action; we hope that this Perspective will help to achieve this. All of us can start by conducting research in a sustainable way. We may also need to change the mode of research that we are comfortable with, yet what better cause can there be than the emergency presented by climate change, which threatens our very existence? The remarkable response to the COVID-19 pandemic has illustrated what can be achieved by investment into molecular life sciences, combined with industrial, political, and societal participation. Let us use this momentum and tackle the challenges of climate change and biodiversity loss head-on.
